# Recent advances in the molecular prognostication of meningiomas

**DOI:** 10.3389/fonc.2022.910199

**Published:** 2023-01-04

**Authors:** Elaina J. Wang, Alexander F. Haddad, Jacob S. Young, Ramin A. Morshed, Joshua P. H. Wu, Diana M. Salha, Nicholas Butowski, Manish K. Aghi

**Affiliations:** ^1^ Department of Neurological Surgery, Brown University, Rhode Island Hospital, Providence, RI, United States; ^2^ Department of Neurological Surgery, University of California, San Francisco, San Francisco, CA, United States

**Keywords:** meningioma, cytogenetics, genomics, epigenetics, methylation, atypical, anaplastic

## Abstract

Meningiomas are the most common primary intracranial neoplasm. While traditionally viewed as benign, meningiomas are associated with significant patient morbidity, and certain meningioma subgroups display more aggressive and malignant behavior with higher rates of recurrence. Historically, the risk stratification of meningioma recurrence has been primarily associated with the World Health Organization histopathological grade and surgical extent of resection. However, a growing body of literature has highlighted the value of utilizing molecular characteristics to assess meningioma aggressiveness and recurrence risk. In this review, we discuss preclinical and clinical evidence surrounding the use of molecular classification schemes for meningioma prognostication. We also highlight how molecular data may inform meningioma treatment strategies and future directions.

## Introduction

Meningiomas account for up to 40% of all primary central nervous system (CNS) tumors, making them the most common primary intracranial tumor (1). While they are thought to be derived from arachnoid cap cells due to cytological similarities (2, 3), the actual cell of origin for meningiomas remains unknown. It is possible they are derived from arachnoid barrier cells, a meningeal cell layer between pia and dura mater separating cerebrospinal fluid from underlying blood vessels, since meningiomas and arachnoid barrier cells have shared expression of prostaglandin D synthase (PGDS) (4, 5). Understanding the origin and natural history of meningiomas is important, since the incidence of meningiomas has been steadily rising, secondary to improvements in imaging resolution and more frequent use of various imaging modalities by providers (6, 7).

The 2021 World Health Organization (WHO) classification system describes 15 meningioma variants categorized into 3 grades based off of histopathological features and molecular biomarkers, with atypical and anaplastic criteria now applied to each of the subtypes (8). Eighty percent of meningiomas fall under the grade 1 category and can be treated with maximally safe resection with the goal of gross total resection (9). However, there remains a 5% 5-year recurrence rate for grade 1 meningiomas that increases to 40% for grade 2 meningiomas (3), and there is no established standard of care (SOC) for grade 2 and 3 meningiomas (10). While meningiomas have been traditionally risk stratified using the World Health Organization (WHO) histopathological grade and extent of surgical resection (8, 11), advances in molecular profiling have highlighted the benefits of utilizing genetic and epigenetic changes to further characterize meningioma aggressiveness and recurrence risk. The inter-observer variability inherent to histopathological diagnoses (12), coupled with advances in genetic and epigenetic technologies that have changed our understanding of meningioma tumor biology, lend support to the need for new molecular classifications for diagnosis and treatment. This review summarizes the use of genetic biomarkers and other forms of molecular data to inform meningioma prognostication and treatment strategies.

## Key genetic changes in meningiomas

### Germline mutations

#### Neurofibromatosis 2

Sporadic mutations in the NF2 gene on chromosome 22 are implicated in 40 to 60% of meningioma patients (3), while 50 to 75% of patients with germline mutations develop meningiomas (13) (**Table 1**). NF2 encodes for the tumor suppressor protein merlin (14), the loss of which is a well-studied driver mutation commonly implicated in high-grade meningiomas (15). NF2 loss-of-function mutations occur through a double-hit mechanism in meningiomas, either through a germline mutation and a second hit with a somatic mutation in syndromic cases, or with a somatic single nucleotide variation or insertion/deletion mutation and an overlapping chromosome 22 deletion event as commonly seen in sporadic cases (15). While 95% of NF2-associated meningiomas remain grade 1 (13), the presence of an NF2 mutation has been associated with increased tumor size and cell proliferation, and it has been suggested that NF2 loss may be the primary and sole initiator of meningioma tumorigenesis in both cranial and spinal meningiomas (16, 17). Two phase II clinical trials (**Table 2**) are currently underway to test FAK inhibitor GSK2256098 and AZD2014 in patients with NF2-mutated meningiomas and NF2 patients with symptomatic meningiomas respectively (19, 20).

**Table 1:** Commonly identified germline and somatic mutations in meningiomas.

Gene	Form of Mutation	Clinical Associations
NF2	Loss of function	Sporadic mutations found in 40-60% of meningioma patients 50-75% of patients with germline mutations develop meningiomas Associated with increased tumor size and cell proliferation
SWI/SNF	Frameshift deletion	ARID1A mutation are independently prognostic of significantly increased hazard of death
KLF4	KLF4 K409 Q missense	The mutation results in upregulation of HIF-1a pathway Meningiomas with this mutation may respond to mTOR inhibition
TRAF7	WD40 domain mutation	TRAF7 mutations are found in nearly one-fourth of all meningiomas Meningiomas harboring TRAF7 mutations tend to be benign, chromosome-stable, and originating from medial skull base
TERT	TERT promoter chr5:1,295,228 (C228T) and chr5:1,295,250 (C250T) regional mutations	TERT promoter mutations are more commonly seen in higher grade meningiomas, particularly WHO grade 3 TERT promoter mutations are associated with significantly shorter time to progression, shorter overall survival, and higher chances of recurrence
AKT1	Gain of function	AKT1 mutations occur with higher frequency among skull base meningiomas and are associated with shorter time to recurrence
SMO/SUFU	Gain of function	Associated with higher recurrence rates among olfactory groove meningiomas Associated with larger tumor volume among anterior skull base meningiomas
PIK3CA	Gain of function	Mutations in PIK3CA are estimated to occur in 7% of non-NF2 mutated meningiomas PIK3CA mutations tend to be mutually exclusive with mutations in AKT1 and SMO
CDKN2A/B	Loss of function mutation	Mutations in CDKN2A/B are associated with shorter time recurrence CDKN2A/B alterations are now included as part of the classification criteria for WHO grade 3 meningiomas
POLR2A	Gain of function mutation	POLR2A-mutant tumors harbor distinct characteristics, including meningothelial histology, and a tendency to originate from tuberculum sellae region POLR2A mutations are found almost exclusively in WHO grade 1 meningiomas

**Table 2:** Phase 2 clinical trials targeting genetic mutations in meningiomas.

Clinical Trial	Duration	Phase	Target	Treatment	Outcome Measures	Reference
NCT02523014	2015-2024	2	SMO, FAK, AKT, CDK	Vismodegib, GSK2256098, Capivasertib, Abemaciclib (n=124)	PFS, CR or PR	Brastianos et al. (16)
NCT02831257	2016-2020	2	mTOR	Vistusertib (n=18)	Radiographic response	Plotkin et al. (17)
NCT03071874	2017-2021	2	mTOR	Vistusertib (n=28)	PFS	Plotkin et al. (18)

PFS, Progression-free survival.

CR, Complete response

PR, Partial response.

#### Switch/sucrose non-fermentable

Germline mutations in two SWI/SNF chromatin remodeling complex subunits have also been implicated in meningioma tumorigenesis: SMARCB1 and SMARCE1 (21). Mutations in SMARCB1 have been linked to the development of multiple meningiomas, while SMARCE1 loss of function mutations have been implicated in patients with familial multiple spinal meningiomas with clear cell histology (22, 23).

#### Suppressor of fused homolog

Germline mutations in known tumor suppressor SUFU on chromosome 10 have long been associated with childhood medulloblastoma, with loss of SUFU leading to disruptions in the sonic hedgehog signaling pathway (24, 25). Germline disruptions in SUFU are also thought to predispose to development of additional cancers such as basal cell carcinoma, gonadal tumors, and meningiomas (26). Mutations in SUFU have been linked to development of isolated familial meningiomas and development of multiple meningiomas (27). In a case series of four related family members with three that had a history of meningiomas, a frameshift mutation in SUFU leading to a premature stop codon was isolated and is posited to be related to development of meningiomas (28).

### Somatic mutations

#### Krueppel like factor 4

KLF4 is a transcriptional regulator known to maintain stemness and found to perform both oncogenic and tumor suppressor functions in a variety of cancers, including but not limited to bladder, esophageal, and gastric cancers (29–32). KLF loss of function has been implicated in colon cancer, while its overexpression has been shown to lead to decreased tumorigenicity of colon cancer cells *in vivo* (33, 34). In meningiomas, it is one of two genes found to be mutated in whole-exome sequencing of sixteen secretory meningiomas (35). KLF4 overexpression in anaplastic meningiomas has been associated with increased expression of tumor suppressor proteins such as p21, p53, and BAX, demonstrating a potential anti-tumor role in higher grade meningiomas (36). Recent *in vitro* data has also suggested that skull-based meningiomas with KLF K409Q mutations have a unique tumor phenotype that may respond to mammalian target of rapamycin (mTOR) inhibition with temsirolimus (37).

#### Tumor necrosis factor receptor-associated factor 7

TRAF7 encodes for a ubiquitin E3 ligase and is the second most commonly mutated gene in meningiomas (38). It catalyzes a variety of ubiquitination reactions, including that of tumor suppressor p53, which has been shown to promote tumor progression in hepatocellular cancer while stabilizing p53’s anti-tumoral effects in breast cancer (39, 40). TRAF7 and KLF4 mutations often co-occur in secretory meningiomas (35), with 40% of TRAF7-mutated meningiomas harboring a KLF4 mutation as well (41). TRAF7 is also one of four genes including KLF4, AKT1, and SMO, likely to be mutated in non-NF2 mutated meningiomas found at the skull base (38). TRAF7 mutations are also closely associated with hyperostosis and often found in spheno-orbital meningiomas (42).

#### Telomerase reverse transcriptase

TERT encodes for telomerase reverse transcriptase, a catalytic subunit of telomerase that promotes cell immortalization *via* telomere elongation (43). Mutations in the chr5:1,295,228 (C228T) and chr5:1,295,250 (C250T) regions of the TERT promoter have been associated with uncontrolled proliferation in several cancers (44–46) and recently in meningiomas that demonstrate histological malignant transformation (47). TERT promoter mutations are more commonly seen in higher grade meningiomas, with mutations found in 1.7%, 5.7% and 20% of 2007 WHO classification grade 1, 2, and 3 meningiomas respectively (18). Clinically, mutations in the TERT promoter region have been associated with significantly shorter time to progression, shorter overall survival, and higher chances of recurrence (18, 48, 49). TERT promoter mutations are now included in the 2021 WHO classification of grade 3 meningiomas (8).

#### AKT1

AKT1 encodes for AKT1 kinase, which regulates cell growth and survival through a variety of pathways (50). AKT1 mutations have been shown to lead to PI3K/AKT pathway activation (51). In a study that applied exome sequencing to 300 meningiomas, mutations in AKT1 were found in 13% of tumors (38). Among skull base meningiomas, AKT1 mutations were found at a higher frequency of 30% and was shown to be associated with shorter time to recurrence (52). In the same study, mutations in AKT1 were found to activate mTOR and ERK1/2 signaling pathways (52). AKT inhibitors have been shown to downregulate the expression of osteoglycin (OGN), an oncogene implicated in meningioma growth, *in vitro* and to stabilize meningotheliomatous meningioma growth in the lung of a patient with multiple intra- and extra-cranial tumors (53, 54).

#### Smoothened

SMO is a G-protein coupled receptor involved in the Hedgehog (Hh) signaling pathway (55). Mutations in SMO have been detected in 3 to 6% of all meningiomas, 28% of olfactory groove meningiomas, and 11% of anterior skull base meningiomas (56–59). Compared to meningiomas with AKT1 mutations, SMO-mutated olfactory groove meningiomas had higher recurrence rates, and when compared to AKT1-mutated or wild type meningiomas, SMO-mutated anterior skull base meningiomas had significantly larger tumor volume (58, 59). Given the targetable nature of SMO mutations, a clinical trial is currently underway to test the SMO inhibitor vismodegib in SMO-mutant meningiomas (19).

#### PIK3CA

PIK3CA encodes for a catalytic subunit of phosphatidylinositol 3-kinase (PI3K) that has been implicated in several human cancers (60). Mutations in PIK3CA are estimated to occur in 7% of non-NF2 mutated meningiomas and tend to be mutually exclusive with aforementioned mutations in AKT1 and SMO (57). In a study assessing 55 meningioma patient samples, PIK3CA mutations were found in two patients who had atypical and anaplastic meningiomas respectively (61). PI3K alterations have also been seen to co-occur with TRAF7 mutations, with these tumors demonstrating lower levels of chromosomal instability and clinical tendencies to arise in the skull base (57). Targeting PIK3CA has long been an area of therapeutic interest given the role that increased protein expression in the PI3K/AKT pathway plays in more aggressive malignant meningiomas (62). Two phase II clinical trials are currently underway targeting the PI3K/AKT pathway with vistusertib and capivasertib respectively (19, 63).

#### CDKN2A/B

The cyclin-dependent kinase inhibitor A and B (CDKN2A/B) gene encodes for three tumor suppressor proteins, the loss of which has been demonstrated to contribute to spontaneous development of melanomas in CDKN2A/B knockout mice (64). In addition to NF2 inactivation, loss of CDKN2A/B contributes to meningioma progression and has been associated with shorter time to recurrence in mice (65). Among a series of 17 recurrent and 13 non-recurrent meningiomas, CDKN2A/B alterations were found only in recurrent meningiomas, and a novel SNV (p.Ala148Thr) was identified in 5 recurrent meningiomas, further supporting the association between CDKN2A/B alterations and meningioma recurrence (66). Along with TERT promoter mutations, CDKN2AB alterations are now included in categorizing grade III meningiomas (8).

#### SUFU

Changes in SUFU lead to dysregulation of the hedgehog (Hh) signaling pathway, the activation of which has been shown to play a critical role in meningioma growth and development, with 72% of Hh signaling pathway genes being differentially expressed in meningiomas compared to normal tissue (67). Across 850 meningiomas that underwent genomic analyses, SUFU mutations were identified in 23 patients and seen to co-occur with PTEN and ARID1A mutations (68).

#### POL2RA

POLR2A, the catalytic subunit of RNA polymerase II, has been shown to harbor mutations that characterize a distinct subset of meningiomas that lack the aforementioned mutations commonly seen in other meningiomas (69). Meningiomas with mutations in POLR2A were exclusively benign with distinct meningothelial histology and were more likely to arise from the tuberculum sellae (69).

### Cytogenetic alterations

There are several copy number variations (CNVs) associated with meningioma pathology (**Table 3**). The initial loss of chromosome 22q in meningioma tumorigenesis has long been established as an early chromosomal event linked to both NF2 mutated and non-NF2 mutated tumors (3, 70). It is estimated to be found in 60 to 70% of all meningiomas, with significantly increased odds among older patients (71, 72). While the loss of 22q occurs in many patients with established neurofibromatosis type 2 syndrome, somatic mutations have also been discovered in around 47% of sporadic meningiomas (73, 74).

**Table 3:** Commonly identified cytogenetic alterations in meningiomas.

Chromosome	Type of alteration	Implication for prognostication
22q	Deletion	Estimated to be found in 60-70% of all meningiomas Both biallelic loss and macro-mutations in 22q are more commonly detected in fibroelastic than in meningothelial histological subtypes
1p	Deletion	Second most common chromosomal event after loss of 22q Loss of 1p has been linked to higher rates of tumor recurrence and progression
14q	Deletion	Third most common cytogenetic change detected among meningiomas Loss at chromosomal arm 14q has also been correlated with increased risk of tumor recurrence
6q, 10q, 17q, 18q, 20q	Deletion	More commonly found in high-grade meningiomas when compared to low-grade meningiomas

In the 40% of meningiomas that do not have NF2 inactivation, alternative explanations are sought to explain meningioma tumorigenesis (75). Losses in chromosomal locations 1p and 14q have been identified in higher grade meningiomas (71). Specifically, loss of 1p is the second most common chromosomal event after loss of 22q, and it has been linked to higher rates of tumor recurrence and progression (76). The frequency of mutations in 1p increases roughly from 30% in grade 1 (2000 WHO classification) tumors to 80% and 100% of grade 2 and 3 tumors respectively (76). Losses in 14q are the third most common cytogenetic change detected among meningiomas, with similar frequencies among WHO grade 1 through 3 tumors as 1p losses (76). Similar to 1p losses, a loss at chromosomal arm 14q has also been correlated to increased risk of tumor recurrence (77).

The concurrent loss of 1p and 3p has also been detected in a small subset of meningiomas without detectable losses in chromosome 22. These losses are hypothesized to contribute to meningioma growth, as changes in chromosome 3 have been linked to other cancers such as breast and small cell lung cancer (78, 79). Other chromosomes such as 6 have been found to harbor genes such as *HIST1H1C* and *CTGF*, changes in which are associated with meningioma recurrence (80). Loss of heterozygosity at certain sites of chromosome 10 have also been predictive of recurrence and worse prognosis in meningioma patients (81).

Greater number of chromosomal anomalies within a tumor has also been associated with higher tumor grade. For instance, in Pfisterer et al., the distribution of chromosomal 1, 14, and 22 anomalies was examined among 77 meningioma cases (82). The loss of 1p, 14q, or 22q alone was only found in grade I meningiomas, while 23% of meningiomas with both 1p and 14q deletions were grade II meningiomas, and 80% of meningiomas with losses in all three chromosomes were grade III (82). In the literature, losses of 1p were found in 75% of high-grade versus 23% of low-grade tumors, 14q losses in 67% of high-grade versus 31% of benign tumors, and chromosomal 22 deletions in 47% of grade II versus 19% of grade I tumors (77, 83). Bi et al. examined the genomes of 39 high-grade meningiomas and found that high-grade tumors were more likely to have loss of chromosome 22 and 1p than low-grade meningiomas, with high-grade meningiomas also commonly exhibiting losses of 1p, 6q, 10q, 18q and gains of 17q and 20q (84).

### Radiation-induced meningiomas

Meningiomas are one of the most common tumors to arise after radiotherapy, particularly in the pediatric population (85, 86). Radiation-induced meningiomas have a tendency to behave more aggressively than sporadic meningiomas and often arise two decades after radiation treatment (87, 88). Unlike sporadic meningiomas that commonly harbor NF2 mutations, the same mutations are not seen in radiation-induced meningiomas (87). Instead, loss of chromosomal segment 1p was found to play a larger role in development of radiation-induced meningiomas, followed by changes in chromosomal locations 9p, 19q, and 22q (87). Among 16 patients with radiation-induced meningiomas, cytogenetic analyses revealed changes in chromosome 1p in 89% of cases and changes in chromosome 6 in 67% of cases (89). Further work exploring the genetics underlying the aggressive behavior of radiation-induced meningiomas may shed light on how to best distinguish benign from malignant sporadic meningiomas.

### Methylation profiling

Epigenetic changes have been found to be useful biomarkers of cancer diagnosis and predictors of recurrence and treatment response. For instance, alterations in hypermethylation of O6-methylguanine DNA methyltransferase (MGMT) in glioblastoma can be useful indicators of chemotherapy response (90). In meningiomas, the absence of trimethylation of H3K27 (H3K27me3) has been shown to be associated with more aggressive growth of tumor, as well as NF2 and SUFU mutations, allowing us to further stratify grade 1 and 2 tumors according to the 2016 WHO classification system (91). In another study examining 1268 meningiomas, the loss of H3K27me3 was found to be a significant predictor of negative prognosis and recurrence, further underlying the importance of methylation profiling in categorizing meningiomas (92).

Methylation profiling has taken on a similar role in identifying and distinguishing meningiomas. When compared to bulk RNA-sequencing, DNA methylation profiling was found to more accurately identify meningioma metastases to the liver in one case report (93). While both bulk RNA-sequencing and DNA methylation profiling could separate the liver metastasis from the primary intracranial meningioma, DNA methylation could better establish the diagnosis of the liver metastasis as a meningioma while hepatocyte-specific gene expression confounded similar findings using bulk RNA-sequencing (93). However, it is important to note that these findings were in the context of one case report, and the improved accuracy of DNA methylation profiling in this instance cannot be generalized. In another study, application of DNA methylation profiling to more than 3000 meningiomas identified an epigenetically distinct cluster of 31 tumors, the majority of which were histopathologically diagnosed as clear cell meningiomas (94). Several studies that have integrated methylation studies with other techniques to classify meningiomas will be discussed in the upcoming section.

## Molecular profiling for meningioma stratification

Advances in computational molecular profiling techniques have allowed for new classifications of meningiomas that account for findings at the DNA level rather than histopathological analysis (95). A variety of techniques including sequencing, methylation profiles, and copy number variation analysis have also been used to generate scores that may better predict prognosis in meningioma patients when compared to standard WHO grading systems.

Genomic sequencing analyses have also been utilized to further classify meningiomas. Patel et al. applied bulk RNA-sequencing and whole-exome sequencing to 160 tumors from 140 meningioma patients to identify 3 classes of meningiomas that were found to predict recurrence more accurately than the standard 2016 WHO grading system (96). Among the three groups (designated type A, B, and C), type C had the highest MIB1 proliferative index, the highest proportion of men, and the shortest recurrence-free survival despite containing primarily WHO grade 1 tumors (96). Type C tumors were also found to have increased expression of FOXM1, leading to loss of the repressive DREAM complex (96). Work by Vasudevan et al. similarly revealed two distinct groups of 280 meningiomas, with aggressive tumors marked by increased expression of FOXM1 (97). FOXM1 has also been implicated as one of three genes upregulated in primary atypical meningiomas, which have also been found to demonstrate NF2 loss, genomic instability, mutations in SMARCB1, and a hypermethylated phenotype (98).

Genomic analyses applied to aggressive meningiomas have also identified 3 distinct groups of meningiomas organized by NF2 status: NF2-mutant, NF2-agnostic, and NF2-wild type (68). NF2-mutant meningiomas were more often associated with men and mutations in CDKN2A/B while NF2-agnostic meningiomas were often associated with TERT and TP53 mutations (68). The third group of NF2-wild type tumors predominantly lacked NF2 mutations and were split further into 3 subgroups: those containing chromatin regulator mutations in BAP1 or PBRM1, skull-based meningiomas with AKT1, PIK3CA, and SMO mutations, and meningiomas with a mix of mutations that shared no discernable pattern (68). Genomic analysis of 300 meningiomas by Clark et al. also revealed benign tumors at the skull base express mutations in TRAF7, KLF4, AKT1, and SMO while higher grade tumors often contained NF2 mutations and were found at the cerebral and cerebellar hemispheres (38).

Genome-wide DNA methylation profiles on 497 meningiomas revealed 6 distinct methylation classes that were found to predict meningioma progression more accurately when compared to 2016 WHO grade 1 tumors and meningioma recurrence more accurately when compared to 2016 WHO grade 2 tumors (99). Similarly, Nassiri et al. applied copy number variation analysis, somatic point mutations, methylation profiles, and messenger RNA abundance to 121 meningioma patient samples to create four molecular groups of meningiomas: immunogenic, benign NF2 wild-type, hypermetabolic, and proliferative (100). The four groups were able to predict patient outcomes more accurately when compared to the existing 2016 WHO classification system and to classification systems based off DNA methylation (100). Methylation analysis by Olar et al. revealed two distinct groups with favorable and unfavorable prognoses respectively (101). Tumors in the unfavorable prognosis group were found to have a higher proportion of copy number aberrations than those in the favorable prognosis group, including losses of 1p and 14q (101).

The integration of histologic findings with genetic profiling including methylation profiling and copy number analysis has further helped improve the precision of meningioma stratification. Using data across 3031 meningiomas, Maas et al. developed an integrated score that predicted risk of meningioma progression more accurately than existing 2016 WHO grading by classifying tumors into low, intermediate, and high-risk groups based off of histology, methylation classes, and CNV analysis (102). While CNV- and methylation-based subtypes independently demonstrated increased prediction accuracy compared to the existing 2016 WHO grading system, the integrated score resulted in further improved accuracy, emphasizing the value of both histology and molecular risk stratification in meningiomas (102).

Integrated use of DNA methylation, RNA sequencing, and cytogenetic profiling by Patel et al. on 110 grade 1 and II meningiomas according to the 2016 WHO classification system revealed two benign and one malignant tumor group closely resembling the previously established type A, B, and C classifications (103). Methylation analysis further distinguished these groups as tumors with balanced methylation (Meth 1), hypomethylation (Meth 2), and hypermethylation (Meth 3) (103). When comparing these groups to those established by Olar et al., Meth 2 and 3 tumors were conferred a favorable and unfavorable prognosis respectively, corresponding with clinical outcomes (101, 103). Further analysis revealed 75% of tumors classified *via* methylation analysis corresponded to a tumor group established by either transcriptional classification (type A, B, C) or cytogenetic classification (no loss, 22q loss, 1p/22q loss) (103).

In a similar vein, Driver et al. created an integrated scoring system by combining chromosomal losses, CDKN2A losses, and mitotic count to separate meningiomas into three separate groups (104). The subsequent grading system reclassified 32% of meningiomas to a higher or low grade when compared to their original WHO grade and was able to more reliably predict risk of recurrence compared to the existing 2007 and 2016 WHO grading system (104). Key molecular alterations associated with high grade included higher mitotic count, 1p del, 3p del, 4p/q del, 6 p/q del, 10 p/q del, 14q del, 18 p/q del, 19 p/q del, and del CDKN2A/B (104). While both Driver and Maas et al. emphasized 22q, 1p, 6q, and 14q loss as the most frequent deletions encountered, Driver et al. also included 3p, 4p/q, 10p/q, 18p/q, 19p/q alterations and mitotic count instead of methylation families in their scoring system (102, 104).

Most recently, DNA methylation profiling on 565 meningiomas was performed and integrated with single cell, proteomic, and other genetic, transcriptomic, biochemical approaches to categorize meningiomas into three distinct clinical groups: merlin-intact meningiomas, immune-enriched meningiomas, and hypermitotic meningiomas (105). Merlin-intact meningiomas were characterized by gain of function in chromosome 5, loss of function in chromosome 6p, and intact NF2 expression, with the best overall survival among the three groups (105). Immune-enriched meningiomas exhibited gain of function in 6p and loss of function in 22q, and notably, lymphocytes in these tumor microenvironments were prone to exhibiting exhaustion markers, potentially explaining why immune checkpoint blockade has not had the same effect on survival in meningiomas as it has in other cancers (106). Hypermitotic meningiomas had the worst overall survival of all three groups and were distinguished by upregulation of FOXM1 expression, which was found to regulate DNA damage response, potentially increasing hypermitotic meningioma resistance to cytotoxic therapy (105).

## Future directions

While the information afforded by integrated scoring systems is an essential first step to guiding meningioma management, many questions remain to be answered particularly regarding adjuvant therapies and post-operative imaging follow-up. For instance, while most studies do not include Simpson grade or gross versus subtotal resection as part of their grading criteria, these are essential factors that guide decisions regarding post-operative management and consideration of radiotherapy. It is important to determine whether there are specific genetic changes that make a tumor amenable to systemic therapies such as upfront radiation versus observation. Since new classification schemes including the recent changes to the WHO grading system may alter how aggressively a tumor is treated, it is also important to determine whether new classification systems are changing outcomes such as recurrence rates.

In addition, with several novel integrated systems of meningioma classification, it will be crucial to compare the accuracy of different systems through further investigation, including cross study group comparisons. Another potential factor limiting the widespread use of novel integrated classification systems is the high cost and limited availability of some of the technologies utilized, including DNA methylation and next generation sequencing. When applying these technologies to categorize meningiomas, it is also important to take note of intratumoral heterogeneity among meningiomas, with spatially distinct areas of the same tumor exhibiting distinct gene expression patterns (107). Moving forward, it is important to determine how the new groupings afforded by integrated scoring systems may change future management of meningiomas.

## Conclusions

Meningiomas are the most common primary intracranial neoplasm and among the most genetically well-studied intracranial tumors. While pre-existing classification schemes by the WHO have traditionally been used to predict meningioma prognosis and risk of recurrence, advances in molecular profiling have allowed for development of several new classification schemes utilizing DNA-level rather than histopathological findings. It is critical to continue applying new sequencing and computational technologies to better predict meningioma behavior in the clinical setting.

## Author contributions

Conceptualization: MA, NB, RM, JY, AH, EW; Writing - Original draft: EW; Writing - revisions: EW, JW; Visualization: AH, EW, DS; Review & editing: MA, NB, RM, JY, AH, JW; Supervision: MA, NB. All authors contributed to the article and approved the submitted version.
